# MicroCT imaging reveals differential 3D micro-scale remodelling of the murine aorta in ageing and Marfan syndrome

**DOI:** 10.7150/thno.26598

**Published:** 2018-11-15

**Authors:** Júlia López-Guimet, Lucía Peña-Pérez, Robert S. Bradley, Patricia García-Canadilla, Catherine Disney, Hua Geng, Andrew J. Bodey, Philip J. Withers, Bart Bijnens, Michael J. Sherratt, Gustavo Egea

**Affiliations:** 1 Departament de Biomedicina, Facultat de Medicina i Ciències de la Salut, Universitat de Barcelona, Barcelona, Spain.; 2 PhySense group, Deptartament de Tecnologies de la Informació i les Comunicacions, Universitat Pompeu Fabra, Barcelona, Spain.; 3 Center for Hematology and Regenerative Medicine, Karolinska Institute, Stockholm, Sweden (current address).; 4 Henry Moseley X-ray Imaging Facility, School of Materials, University of Manchester, Manchester, United Kingdom.; 5 Geotek Ltd, Daventry, United Kingdom (current address).; 6 Division of Cell Matrix Biology & Regenerative Medicine, School of Biological Sciences, University of Manchester, Manchester, United Kingdom.; 7 Diamond Light Source, Oxfordshire, OX11 0DE, Oxford, United Kingdom.; 8 ICREA, Barcelona, Spain.; 9 Institut d'Investigacions Biomèdiques August Pi i Sunyer (IDIBAPS), Barcelona, Spain.; 10 Institut de Nanociència i Nanotecnologia (IN2UB), Universitat de Barcelona, Barcelona, Spain.

**Keywords:** Marfan syndrome, microCT, aorta, ageing, elastic lamella

## Abstract

Aortic wall remodelling is a key feature of both ageing and genetic connective tissue diseases, which are associated with vasculopathies such as Marfan syndrome (MFS). Although the aorta is a 3D structure, little attention has been paid to volumetric assessment, primarily due to the limitations of conventional imaging techniques. Phase-contrast microCT is an emerging imaging technique, which is able to resolve the 3D micro-scale structure of large samples without the need for staining or sectioning.

**Methods:** Here, we have used synchrotron-based phase-contrast microCT to image aortae of wild type (WT) and MFS *Fbn1*^C1039G/+^ mice aged 3, 6 and 9 months old (n=5). We have also developed a new computational approach to automatically measure key histological parameters.

**Results:** This analysis revealed that WT mice undergo age-dependent aortic remodelling characterised by increases in ascending aorta diameter, tunica media thickness and cross-sectional area. The MFS aortic wall was subject to comparable remodelling, but the magnitudes of the changes were significantly exacerbated, particularly in 9 month-old MFS mice with ascending aorta wall dilations. Moreover, this morphological remodelling in MFS aorta included internal elastic lamina surface breaks that extended throughout the MFS ascending aorta and were already evident in animals who had not yet developed aneurysms.

**Conclusions:** Our 3D microCT study of the sub-micron wall structure of whole, intact aorta reveals that histological remodelling of the tunica media in MFS could be viewed as an accelerated ageing process, and that phase-contrast microCT combined with computational image analysis allows the visualisation and quantification of 3D morphological remodelling in large volumes of unstained vascular tissues.

## Introduction

Aortic wall components are highly structured in order to confer the elasticity needed for dampening the stroke pressure and propelling the blood downstream 1,2. Mutations in the genes encoding elastic lamellae proteins can perturb biomechanical and/or biochemical homeostasis, which in turn can lead to vascular pathologies 1,3. In the case of fibrillin-1, mutations induce both aberrant developmental assembly of the lamellae and an increase in bioactive TGF-β in the mature animal 4,5. These alterations cause the relatively common (1:5,000 individuals) multisystem disorder Marfan syndrome (MFS; OMIN #154700), which typically manifests cardiovascular (thoracic aorta aneurysm), ocular (ectopia lentis), and musculoskeletal system (tall stature with arachnodactyly) alterations 6. The irreversible pathological dilation of the aorta usually leads to life-threatening dissections and ruptures 7. Crucially, aortic aneurysms (both thoracic and abdominal) also occur in the absence of fibrillin-1 mutations and are a major health concern in the ageing population of developed countries due to their high incidence (4.8% of the population will develop an aneurysm during their lifetime) and potentially fatal outcome. Currently, the most effective treatment is invasive surgery 8,9. In order to prevent aneurysms and/or develop less invasive treatments, it is of great importance to characterise the progressive micro-structural remodelling of the vessel wall because of both heritable disease and age.

Historically, the study of vascular tissue structure has relied primarily on the microscopic visualisation of two-dimensional (2D) sections by either optical or transmission electron microscopy. These methods have clearly identified extensive remodelling of the vessel wall including fragmentation of elastic lamellae and loss of vascular smooth muscle cells in the tunica media 9,10,11. However, despite their wide use in pathological diagnosis, these techniques are prone to inducing artefacts (primarily because of mechanical sectioning). Additionally, they are not well suited to resolving the three-dimensional (3D) structure of substantial tissue volumes, although serial sectioning (combined with optical or electron microscopy), serial block-face scanning electron microscopy, and confocal microscopy may be used to visualise 3D micro- and nano-structures of small tissue volumes 12,13. Compared with these approaches, X-ray computed micro-tomography (microCT) can visualise the 3D structure of relatively large samples (up to cm^3^) at high resolutions (sub-µm) 14. This non-destructive technique has been applied to cardiovascular samples 15-17, and can image internal tissue structures in unstained cardiovascular tissues using synchrotron-based phase-contrast microCT 18-20. In particular, Stergiopulos and coworkers 21 used microCT imaging at low resolution to map aortic tissue for subsequent histological analysis, whilst Assemat *et al.* 22 characterised aortic geometry for numerical simulations. Finally, we have shown 15 that phase-contrast microCT (which does not rely on exogenous contrast agents) can quantify differences in the sub-micron structure of rat carotid arteries as a consequence of intra-luminal pressure and, using a high-flux synchrotron X-ray source, collagen orientation within the annulus fibrosus of native (unfixed and unstained) intervertebral disc 23. This technique is becoming more popular in cardiovascular research 20,24,25, probably because of its potential clinical relevance 26. However, despite the potential of high-resolution microCT imaging to visualise the internal micro-structure of blood vessel walls 15, it has not been applied to characterise 3D remodelling as a consequence of MFS-associated aneurysms and/or ageing.

In the present study, we demonstrate the capability of synchrotron-based phase-contrast microCT to image intact, large volume vascular structures at micrometre scale resolutions. We acquired microCT scans of WT and MFS mice aortae of different ages and developed new image processing and analysis procedures to evaluate key histological features in 2D and 3D. Using this technique, we were able to characterise similarities and differences between ageing- (from 3-9 months) and MFS-associated remodelling in the aortic wall.

## Methods

### Experimental animals and sample preparation

Three-, six- and nine-month-old MFS (*Fbn1*^C1039G/+^) mice 27 and age-matched WT littermates were used in this study (n=5 mice for each experimental group giving a total of 30 animals, whose aortae were subsequently imaged and analysed). The University of Barcelona's Ethical Committee for Animal Welfare (CEEA) approved the animal care and experimental procedures according to the University of Barcelona's guidelines and the European Parliament Directive. The mice were on a C57B/6 genetic background and maintained as a heterozygous breeding colony in our animal room facility. Animals were sacrificed by isoflurane inhalation, and their aorta was surgically harvested from the aortic root to the suprarenal portion. Samples were immediately rinsed in PBS and fixed in 4% paraformaldehyde. Thereafter, aortae were dehydrated and embedded in paraffin for conventional histological preparations. Sample paraffin blocks were manually trimmed to remove embedding surplus surrounding the aortic vessel, so they finally had a candy cane shape. Aortic paraffin blocks were glued vertically to a magnetic CryoCap (Molecular Dimensions Limited, UK), to provide standing stability once at the microscope setup (**Figure S1**).

### Synchrotron X-ray phase-contrast micro-tomography

Aortic paraffin blocks (n=30) were imaged at the Diamond-Manchester Branchline I13-2 of the Diamond Light Source (Didcot, Oxford, United Kingdom) 28,29 by synchrotron phase-contrast X-ray micro-tomography (microCT) 15. An illustrative image of the experimental set-up is shown in **Figure S1**. Samples were scanned using a wide wavelength spectrum beam (pink beam, 5-35 keV), and the undulator gap was set to 5 mm for data collection. Images of 0.14 s exposure time were acquired with a pco.4,000 camera (PCO AG, Germany) mounted on a scintillator-coupled microscope, placed at a propagation distance of 200 mm from the sample. Each scan comprised 3001 projections collected over 180° of continuous sample rotation at 8× total magnification, providing a 4.5×3 mm field of view and an isotropic 1.1 µm voxel size. The region of interest of the sample was placed close to the centre of rotation to minimise imaging artefacts. The scan time was 7 min per sample. Synchrotron scans were reconstructed via filtered back projection, with flat and dark correction and ring artefact suppression, without Paganin filtering, using DAWN 30,31. The reconstructed tomographic volume image stacks were composed of 2,671 TIFF images of 4,008×2,672 pixels in 32-bit greyscale.

### Computational processing for aortic image segmentation

Aortic volume image stacks were processed in Avizo software (FEI, Thermo Fisher Scientific) to generate a 3D volume-rendered image of the whole aortic vessel by segmenting tissue from paraffin by means of greyscale thresholding. The stacks were virtually rotated and resampled to obtain an image stack orthogonal to the ascending aorta tube, yielding perfect transverse virtual cuts of this vessel region. Finally, stacks were saved as 16-bit unsigned TIFF format. When necessary, image artefacts (contamination or air bubbles) were manually erased by painting in ImageJ 32.

For morphological quantification of the aortic wall in the transverse perspective, a central 200-images-portion of the ascending aorta stack was selected in all the samples. This cropped image stack was visualised and quantified by an in-house graphical user interface (GUI) developed in Matlab (2016, The MathWorks Inc., Natick, Massachusetts, USA), which performed image processing and subsequent histological parameter quantification. Briefly, each image was automatically binarised to segment the aortic wall from the surrounding external paraffin. Several morphological operations were applied to smooth the segmentation (please see the supplementary methods for a detailed description of each processing step). The application of these steps to segment the aortic wall and lumen and then the elastic lamellae are illustrated in **Figure S2** and **Figure S3**, respectively. In order to subsequently segment the tunica media from the adventitia, a first media-adventitia limit was automatically calculated by the GUI. The thickness of the aortic wall was computed in all the image slices by calculating the Euclidean distance transform of the binary mask and by defining a line that defined the middle of the aortic wall throughout the circumference (this line was placed equidistant from both the wall-lumen and the adventitia-paraffin limits, so that it divided the aortic wall into two halves). Subsequently, the limit between media and adventitia layers was determined by dilating the mask that enclosed the inner half of the aortic wall, with a structural element of 0.35×minimum thickness of the aortic wall. This size of the morphological dilation was defined after visual inspection of several images, as it approximately fitted the real media-adventitia limit for all the different aortae sample groups. Importantly, whilst this factor value was applicable to C57B/6 mice at 3, 6 and 9 months old, its suitability for other strains and ages of mice is unknown.

**Figure 1:**
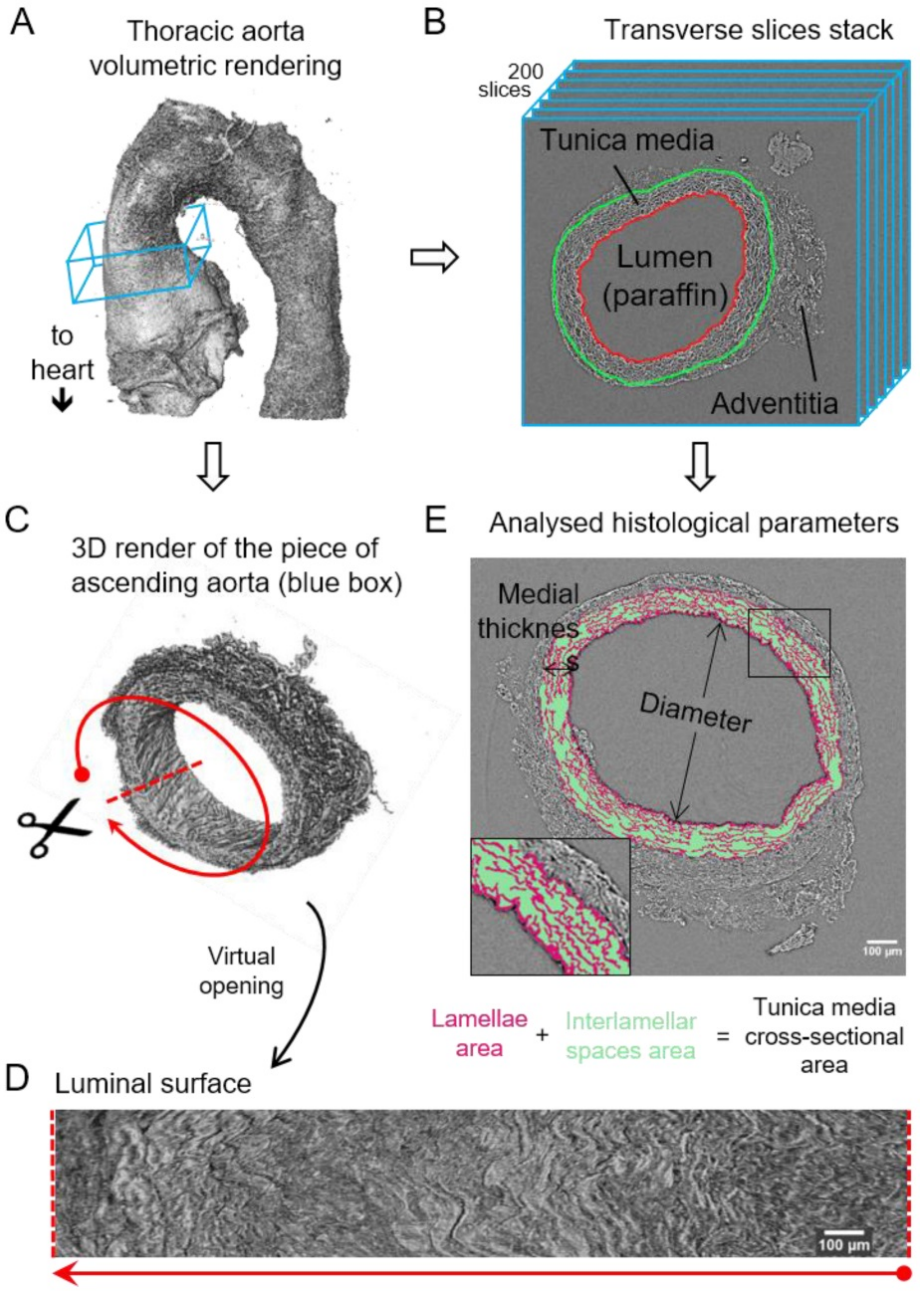
** Image processing steps for aorta microCT scans. (A)** Volumetric rendering of aorta scan from the aortic root to descending thoracic aorta. The blue box includes a slice stack of the ascending aorta indicated in B. **(B)** Representation of the stack made of 200 transverse slices from the ascending aortic wall. Red line traces the luminal surface and green line the media-adventitia limit. **(C)** Volumetric rendering of the 200 slices from the ascending aorta stack (blue box in (A)). Scissors represent the virtual opening process performed by the code. **(D)** IEL luminal surface example obtained after the virtual opening of the ascending aorta. **(E)** Display of the histological parameters quantified in the transverse aortic slices. Scale bars in (D-E), 100 µm.

The centre of the aorta was calculated by performing a spline interpolation of all the lumen centroids computed in all the image slices. After automatically finding the centreline, a local Cartesian to Polar coordinates transformation was applied in order to virtually open the aorta and hence straightening the aorta (the aortic wall converted from a ring into a straight line) (**Figure 1C-D**). This was achieved by using each point of the limit between the lumen and the aorta wall as a local centre for the coordinate values swap. The polar coordinates of the calculated media-adventitia limit were smoothed using a spline function. The GUI enabled the user to modify the limits manually in order to improve the automatic estimation of the media-adventitia limit. It is noteworthy that it was necessary to modify only 2 slices per sample out of the 200 being measured since the GUI readjusted the rest of the images relying on the manual modifications. In summary, the media-adventitia and lumen-aorta limits were automatically found by the GUI, which allowed the generation of tunica media, aortic wall and lumen masks. The GUI code is freely available upon request to G.E. (gegea@ub.edu) or P.G-C (patricia.garciac@upf.edu).

### Computational measurement of morphological parameters

Once the media-adventitia limit was accurately delineated, the thickness of the tunica media, its cross-sectional area and the area occupied by lamellae were computed. The thickness was obtained by calculating the Euclidean distance between the media-adventitia and the media-lumen limits in each image slice. The distribution of all measured thickness values in each virtual slice was plotted as a histogram and then fitted to a Gaussian, and thus, the mean thickness and standard deviation were calculated for all slices in the stack. Cross-sectional area was calculated as the area of the tunica media mask. Subsequently, the medial mean thickness and mean cross-sectional area of the whole 200-slices stack and their standard deviations (SD) were obtained. To calculate the lamella area, the “high contrast lamellae” were automatically segmented out by greyscale thresholding the tunica media images, as their brightness was higher than the rest of the image. More details about the automatic segmentation are given in supplementary material and illustrated in **Figure S2** and **Figure S3**. Thereafter, the total number of pixels representing the lamella was counted and divided by the area of the tunica media to compute the % of medial space occupied by lamellae. Subtraction of lamellar area from the total medial area yielded the area occupied by the interlamellar spaces. Lumen diameter measurements were obtained by approximating the vessel internal circumference to an ellipse, and averaging its major and minor axes (to mimic the way in which the aortic diameter is assessed by echocardiography in clinical and preclinical studies 33,34).

In the case of abnormally dilated samples, the GUI was not able to perform the media-adventitia limit computation nor the virtual opening step, since the vessel circumference was not circular. To overcome this issue, the brightest parts of the images (mainly lamellae) were thresholded and then a watershed algorithm was applied to the resulting binary mask in ImageJ, thus obtaining a mask of a closed tube. Next, the ilastik software 35 was used to identify and segment the lumen from all 200 slices by means of the carving workflow. The resulting lumen mask was applied to the original images in order to create an aortic image stack with a black lumen instead of with a grey lumen. Then, the media-adventitia limit was manually outlined to allow segmentation of tunica media from the rest of the image. Using this approach, the in-house GUI was able to calculate the tunica media cross-sectional area and pixel amount, but not the thickness (due to the irregularities in the vessel circumference). Therefore, the medial thickness of just two irregular samples was manually measured using ImageJ. A preliminary comparison of medial thickness measured either by our automated objective methods or manually using subjective judgement gave similar results (data not shown).

The internal elastic lamina (IEL) luminal surface of all the microCT scans (n=30) was obtained for all the whole ascending aorta region, which included a different number of image slices depending on the sample (from 200 to 800 slices). This image stack corresponding to this region was virtually opened and lumen and tunica media were labelled, following the previously described procedure using the GUI. Next, using an in-house ImageJ macro, we were able to delineate the border between the lumen and the aortic wall, which corresponds to the location of the IEL. Binary dilatation of this limit by 7 pixels was optimal to segment the entire IEL. Finally, we resliced the segmented IEL stack to the en-face perspective and generated the maximal projection of all the images to obtain the visualisation of the IEL luminal surface. Quantification of lamella breaks in the maximal projection image was done manually, delimitating their contour and measuring their area in ImageJ.

### 3D volume rendering

The 3D volume render of aortic tissue was generated using Blender software after manually processing the images with ImageJ. In brief, a small portion of the entire aortic scan was selected, and an intensity threshold was applied to obtain the mask of the lamellae. Following manual smoothing and cleaning of the mask, a contour mesh was generated for the mask stack in Paraview software 36, rendering a lamellae 3D volume of the aortic tissue piece. Next, the number of mesh faces was reduced using Instant meshes software. Finally, the volumetric mesh of the lamellae was visualised by Blender software and a movie was recorded.

### Statistics

Data were analysed using GraphPad Prism 6 (La Jolla, California, USA) and plotted as the 5 data points corresponding to the mean value of each sample and the group mean ± SD. Since the data did not follow a Gaussian distribution and presented with variable distributions, statistical analysis was carried out using the Kolmogorov-Smirnov nonparametric test. An F-test was applied to evaluate differences in data dispersion between groups instead of evaluating differences in the mean. The degree of significance was *p≤0.05, **p≤0.01, and ***p≤0.001. Some plots also contain # to indicate p values of 0.05-0.08, which suggest a trend that would be significant in larger cohorts.

## Results

### Volumetric and image processing of aortae tomograms

In total, 30 aortae (from WT and MFS mice at age 3, 6 and 9 months old [mo]; n=5 per group) were fixed, dehydrated and paraffin embedded. Next, we imaged these samples by synchrotron-based phase-contrast microCT without staining or sectioning (this approach was similar to that employed for a laboratory X-ray source as described by Walton *et al.* 15). Thereafter, we applied a newly developed automatic image processing protocol embedded in a user-friendly GUI (available upon request as detailed in Methods) to quantify key structural features of the aortic wall in the tomograms acquired by microCT (**Figure 1**).

**Figure 2:**
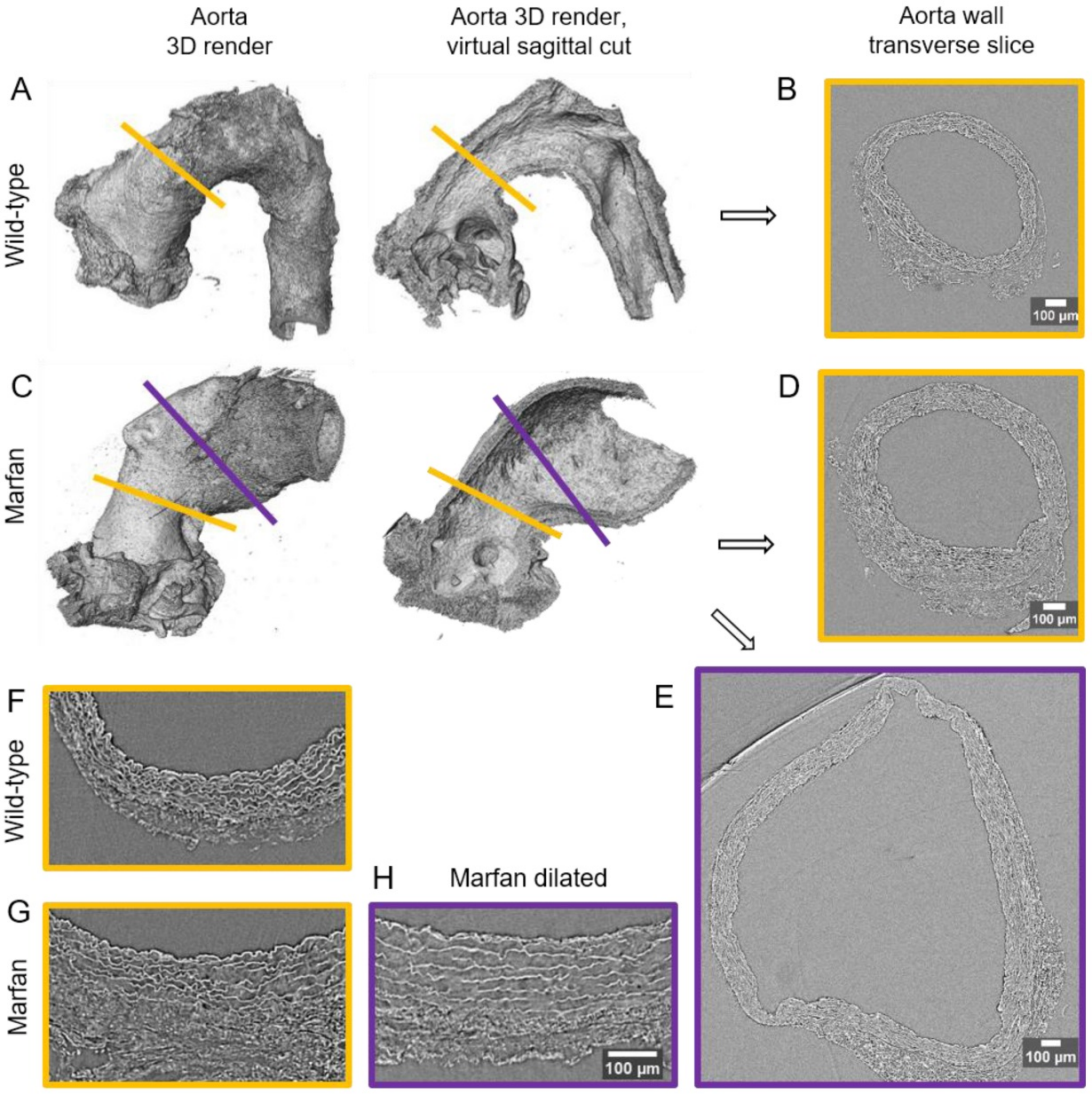
** Synchrotron-based microCT scan examples of wild type and Marfan syndrome mice aortae. (A)** Wild-type aorta volumetric rendering. **(B)** Transverse slice of wild-type aorta corresponding to the yellow line in (A). **(C)** Volumetric rendering of a Marfan aorta with a non-dilated (yellow line) and a dilated (purple line) zones. **(D)** Transverse slice of the Marfan aorta at the non-dilated zone (yellow line in (C)). **(E)** Transverse slice of the Marfan aorta at the dilated zone (purple line in (C)). **(F-H)** Representative transverse sections from microCT scans of 9 month-old wild type and Marfan mice aortae (dilated and non-dilated zones). Scale bars in (B, D-E, H), 100 µm.

The resultant phase-contrast tomograms are virtual volumes (**Figure 1A**), which can be sliced in any orientation, just like clinical CT scans. Slices extracted transverse to the vessel axis clearly revealed internal anatomical structures, which mapped closely to conventional histological features including the lumen, tunicae media (and constituent elastic lamellae), outer adventitia and supporting paraffin (**Figure 1B**). As the tunica intima did not produce enough phase contrast to be visualised by microCT, the internal elastic lamina (IEL) constituted the border between luminal paraffin and aortic wall in the slices (red line in **Figure 1B**). From the virtual volumes, the interior surface of the vessel can be visualised, disclosing characteristic wrinkles caused by loss of physiological intra-luminal pressure (central images in **Figure 2A, C**). This interior surface can be computationally extracted by virtually opening the IEL to visualise its luminal surface (**Figure 1C-D**). For quantitative analysis of internal wall structure, we extracted an aortic ring (blue box in **Figure 1A**) composed of 200 consecutive slices (**Figure 1B**) at the same anatomical position in all the ascending aortae of the examined groups. Ascending aorta lumen diameter, the thickness and cross-sectional area of the tunica media, and the cross-sectional area of the elastic lamellae and interlamellar spaces were quantified (**Figure 1E**). Some aortic samples showed a clearly visible bulging in the tubular portion of the ascending aorta (**Figure 2C**), which most likely is indicative of the aneurysm. However, it cannot be ruled out that such bulges may be also caused or exacerbated by other non-genetic factors since aortae were processed in an unpressurized state. Therefore, hereafter we will refer to these bulges as dilations, and the bulging regions as dilated zones.

### MicroCT-based histopathological aortic wall remodelling analysis in middle-aged wild-type and Marfan mice

**Figure 3:**
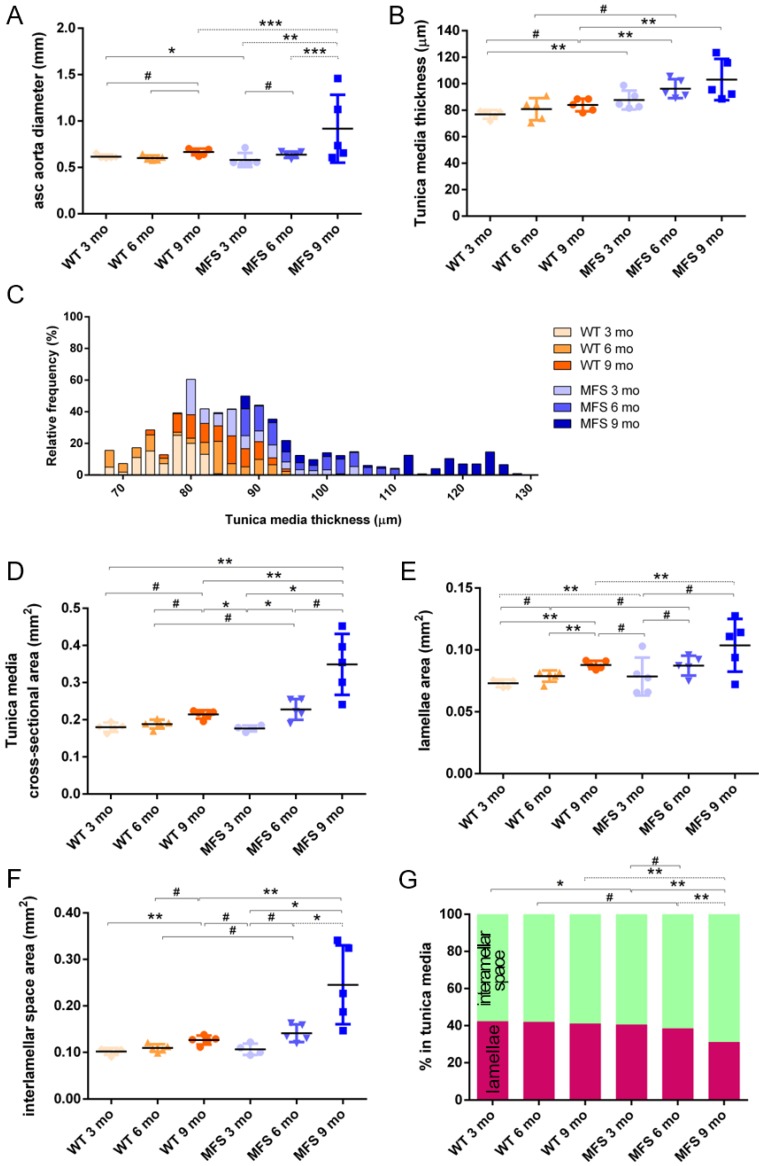
** Transverse histological parameters in aged wild type and Marfan syndrome mice. (A)** Ascending aorta luminal diameter (unpressurised). **(B)** Tunica media thickness mean values of each aorta. **(C)** Frequency distribution of the 200 thickness values per aorta. **(D)** Tunica media cross-sectional area. **(E)** Tunica media area occupied by lamellae. **(F)** Tunica media area occupied by the interlamellar space. **(G)** Tunica media percentage composition. WT: wild-type mice; MFS: Marfan syndrome mice. Results are mean ± SD. Continuous line corresponds to a mean statistical test, and dashed line to a dispersion statistical test.

**Figure 4:**
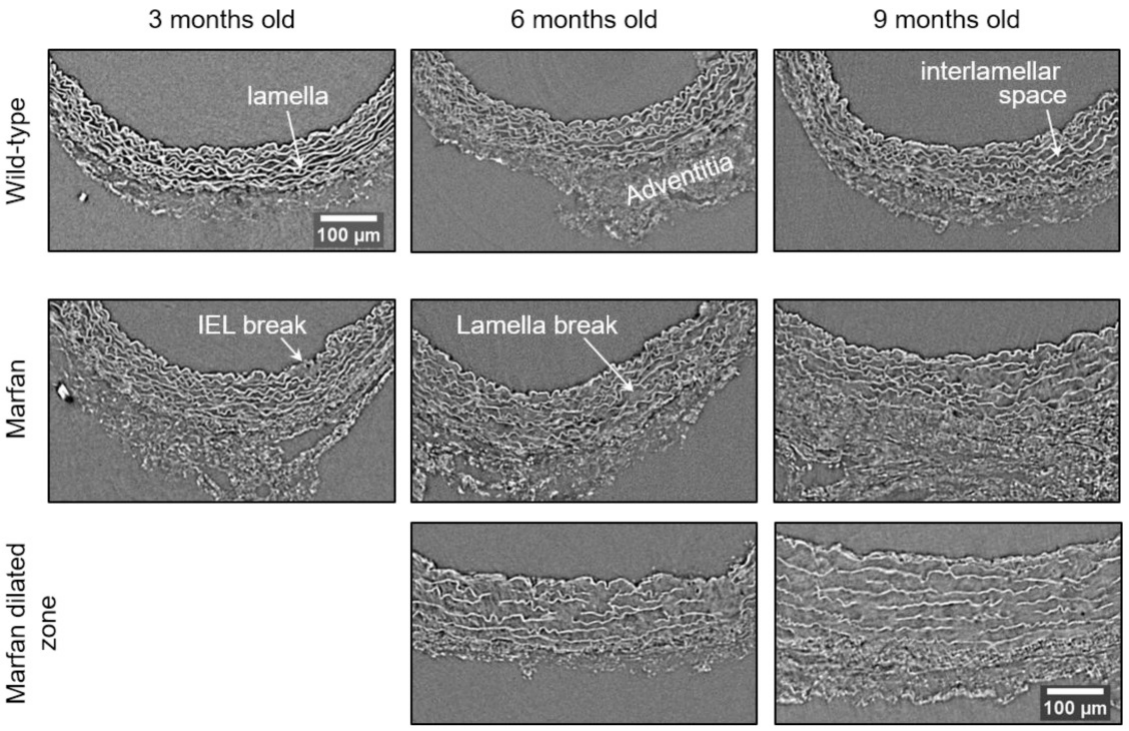
** Representative transverse sections from microCT scans of wild-type and Marfan syndrome mice aortae at 3, 6 and 9 months old.** IEL: internal elastic lamina. Scale bar, 100 µm.

In WT mice, all aortae showed a regular candy-cane shape (**Figure 2A**) and the ascending aorta conduit was circular or slightly ellipsoid (**Figure 2B**) with an average diameter of 0.6±0.04 mm (**Figure 3A**). The mean diameter of the ascending aorta (**Figure 3A**) and the thickness of the tunica media were not significantly different with age but consistently increased (# in **Figure 3B**; 3 mo = 77±4, 6 mo = 81±8, 9 mo = 84±5 µm). However, this analysis does not take into account the richness of these microCT data sets, which characterise anatomical variation along the vessel axis. Comparing the frequency distributions of media thickness measurements for each slice from each animal (n=1,000 slices per experimental group) there is a clear shift towards greater increased media thickness with age (**Figure 3C**), and this result is not due to the inclusion of outlier animals given the tightness of the distributions (notice the low SD) (**Figure 3B**). Hence, results indicate that both WT diameter and media thickness values tend to increase with age. In addition, the mean cross-sectional area of the WT tunica media had also a strong tendency to increase with age (# in **Figure 3D**). This age-associated remodelling of the WT tunica media was due to significant increases in the cross-sectional areas of both the lamellae and the interlamellar spaces (**Figure 3E-F**, respectively). Since both tunica media constituents (lamellae and inter-lamellar spaces) augmented at the same rate, their medial percentage remained constant with age (**Figure 3G**). Representative transverse images of aorta wall at different ages are shown in **Figure 4**.

The same histological parameters were analysed in MFS aortae (**Figure 2** and **Figure 3**). As with WT mice, MFS diameter, media thickness and cross-sectional areas also increased with age. However, all histological parameters values measured in MFS 9 mo mice were rather heterogeneous (dark blue datasets in **Figure 3A-F**), probably because three of these aortae developed evident dilations (**Figure 2C**). Therefore, comparison of dataset dispersion (by statistical F-test; dashed lines in the plots) instead of the conventional mean values (by Kolmogorov-Smirnov non-parametric test; continuous lines) was key to compute statistical differences for this highly variable experimental group (**Figure 3**). Taking this into account, comparative analysis between the three age stages of the MFS condition showed: (i) a significant increase in the ascending aorta luminal diameter (**Figure 3A**); (ii) a tendency to augment the media thickness (**Figure 3B-C**); (iii) a significant enlargement of the tunica media cross-sectional area (**Figure 3D**); and (iv) a significant, gradual decrease of the percentage of medial space occupied by lamellae (**Figure 3G**). Importantly, this latter change was not caused by a reduction in lamellae area (**Figure 3E**), but by a significant two-fold increase of the interlamellar space area (**Figure 3F**). Considering WT and MFS datasets together, MFS 9 mo aortae displayed significantly higher values than same age WT specimens in all the quantified histological parameters: MFS 9 mo had both a larger diameter and thicker tunica media, and greater lamellae and interlamellar space cross-sectional areas than WT 9 mo (**Figure 4**). MFS tunica media thickness and lamellae percentage area were already significantly greater at age 3 mo compared to WT (**Figure 3B, G**), whereas at 6 mo these parameters showed a strong tendency to be higher than in the WT (**Figure 3D-F**). Finally, the tunica media thickness in MFS 6 mo aortae was significantly higher than that in WT 9 mo vessels (**Figure 3B**). Representative images of this histopathological remodelling in MFS aortae are shown in **Figure 4**.

**Figure 5:**
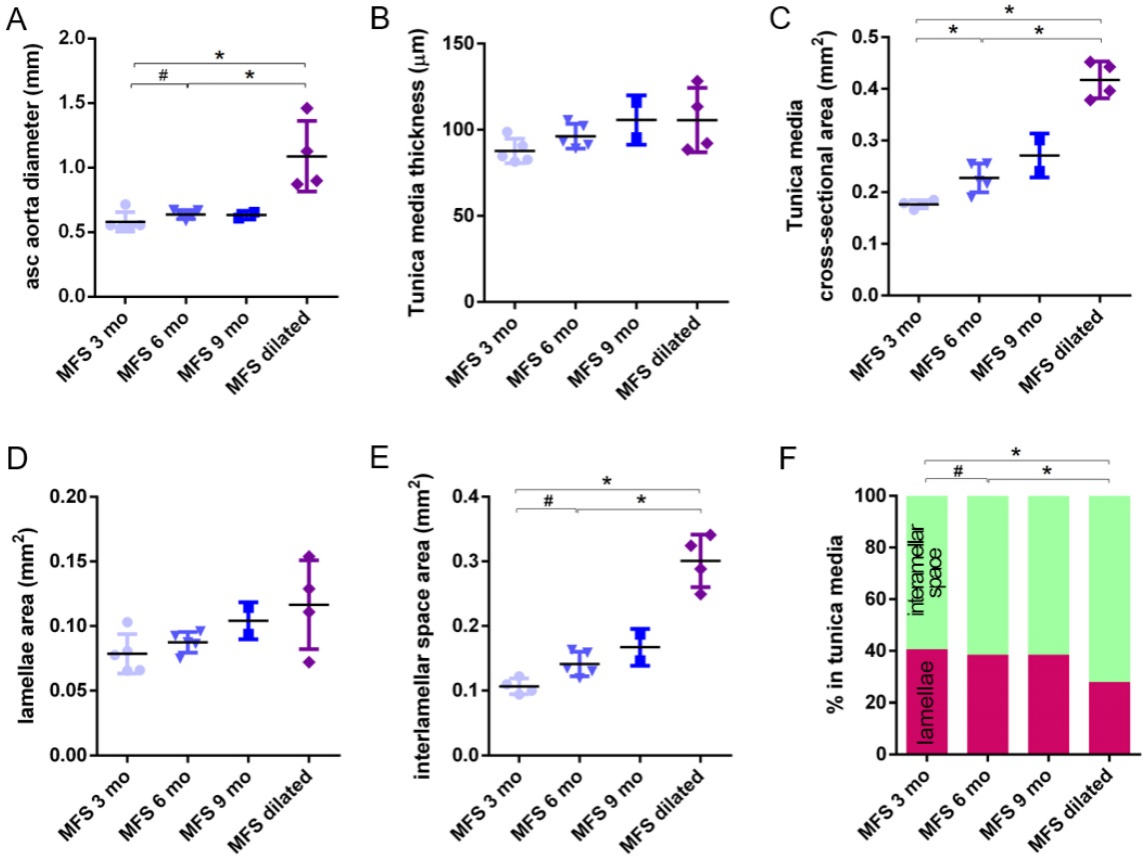
** Transverse histological parameters in dilated and non-dilated aged Marfan syndrome mice aortae.** Anatomically evident MFS dilated aortic zones were categorized separately (purple data here, and purple line in **Figure 2**), resulting in MFS 9 mo with n=2, and therefore no statistical comparisons were performed with this group. **(A)** Ascending aorta luminal diameter (unpressurised). **(B)** Tunica media thickness mean values. **(C)** Tunica media cross-sectional area. **(D)** Tunica media area occupied by lamellae. **(E)** Tunica media area occupied by the interlamellar space. **(F)** Tunica media percentage composition. WT: wild-type mice; MFS: Marfan syndrome mice. Results are mean ± SD.

Clear ascending aortic dilations that had an adjacent non-dilated zone were evident in one MFS 6 mo and one MFS 9 mo mouse (**Figure 2C**, yellow line for non-dilated, purple line for dilated). Additionally, two MFS 9 mo mice showed large aortic wall dilations that occupied the whole ascending aorta (hence no adjacent non-dilated zone was evident). As the quantitative analysis was performed on the same ascending aorta region for all samples, the MFS 9 mo group included measurements at “normal” non-dilated zones from three animals, and measurements from the two entirely dilated aortas. Therefore, in order to evaluate more in detail the impact of dilation occurrence on the remodelling of the MFS aortic wall, the analysis of the histological parameters was subsequently categorised into non-dilated (MFS 3, 6 and 9 mo in **Figure 5**) and dilated (MFS dilated in **Figure 5**) groups. Thus, the histological parameter values of the two entirely dilated aortas previously assessed in the MFS 9 mo group (dark-blue set data in **Figure 3**) were now transferred to the MFS dilated group (purple data set in **Figure 5**), and additional measurements were made at the dilated zones with adjacent non-dilated ones to complete the MFS dilated group. Comparative analysis of this new MFS dataset categorisation revealed that the diamater in these dilated zones was clearly greater than those in the other MFS groups (**Figure 5A**). Likewise, tunica media cross-sectional area was also significantly increased in MFS dilated compared to the other MFS groups (**Figure 5C**) as was the interlamellar space area (**Figure 5E**). However, the tunica media thickness and the lamellae area remained similar in all MFS groups (**Figure 5B, D**). Consequently, lamellar area was signficantly lower in dilated regions of MFS vessels (**Figure 5F**). This structural remodelling can be clearly seen in the aortic wall images (**Figure 4**). Subtraction of the dilated samples from the MFS 9 mo group left the dataset with only n=2, and consequently no statistical comparisons with this group were performed. Nevertheless, we observed that this dataset transference from MFS 9 mo to MFS dilated abolished the previously observed trend for increased tunica media thickness (compare **Figure 3B** vs. **Figure 5B**). Moreover, the dataset transference revealed that dilated samples were responsible for the highest tunica media cross-sectional area and interlamellar space area values. This lead to an increasing tendency of both parameters that was progressive from MFS 3 mo to MFS dilated group (**Figure 5C, E**). In addition, the lamellae area was highly variable in MFS dilated samples (**Figure 5D**).

### Three-dimensional micro-scale injuries in the tunica media of aged and Marfan aortae

**Figure 6:**
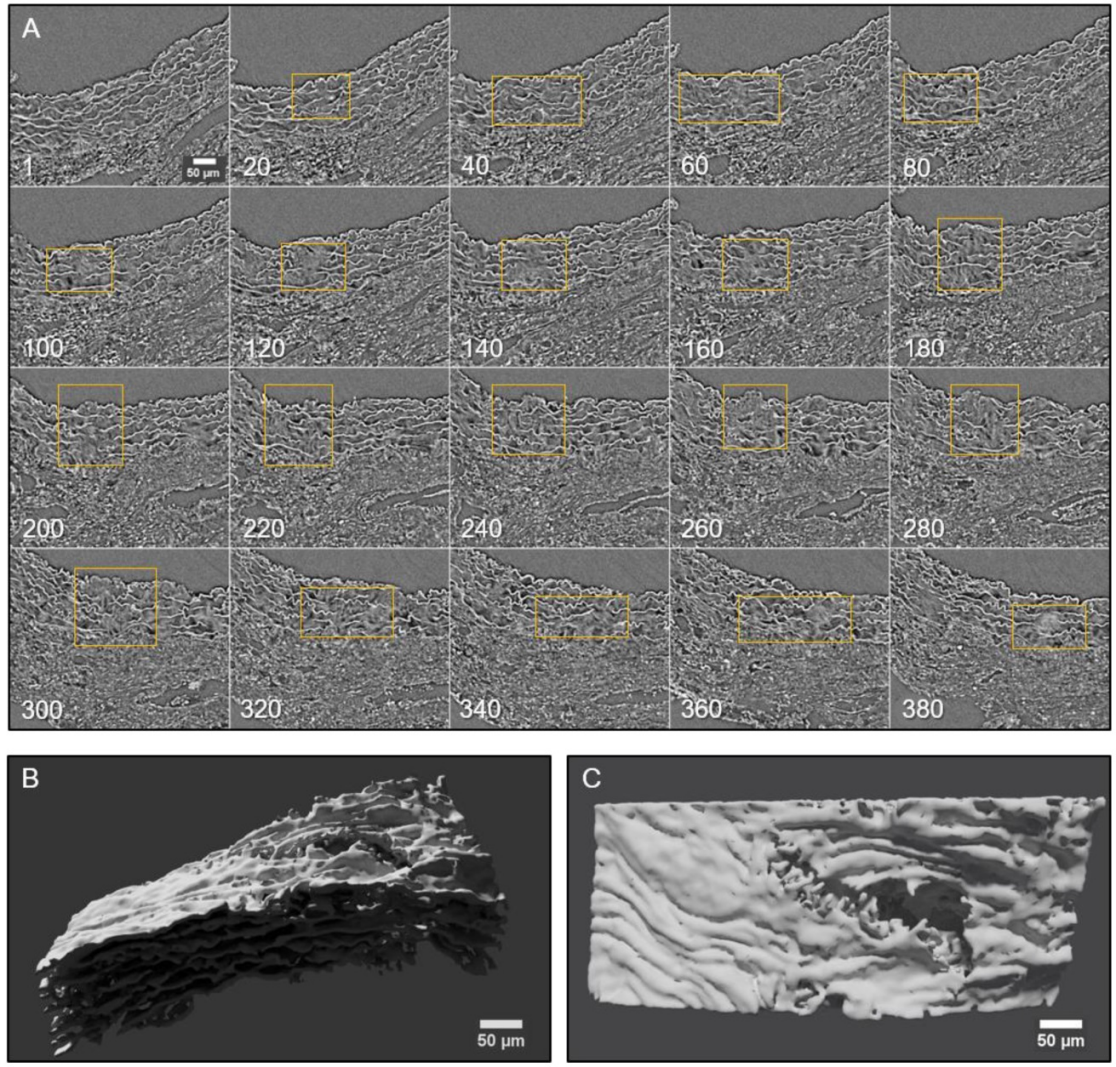
** 2D and 3D visualisation of lamellae breaks progression in the tunica media of a Marfan syndrome aorta**. **(A)** Transverse slices sequence of Marfan mice aortic wall. The yellow box marks the same break progressing both axially and laterally. In the beginning, there is a break in one or two lamellae, whose damage gradually worsens, affecting several neighbouring lamellae (slice 140) and the IEL (slices 240 to 300). **(B-C)** Volumetric rendering of the same aortic wall piece shown in (A) viewed laterally (B) or en-face (C). Here, only lamellae of the tunica media are rendered. Notice the extension and depth of the break, and the impact to multiple lamellae to different extents. Scale bar, 50 µm.

Conventional histological images (2D transverse sections) of MFS aortic media typically show elastic lamellae breaks (discontinuities in the magenta-coloured lamellae in **Figure 1E** and **Figure 4**). However, these breaks are unlikely to be confined only to the axial region visualised in the section. Many breaks will extend into preceding and/or subsequent sections. Since sampling at different positions of a same specimen is rarely performed in conventional histological studies, the axial extension of these lamellae breaks and hence their 3D shape is currently poorly defined. In contrast, microCT has the potential to characterise the volumetric morphology of tissues by providing the sequence of contiguous virtual sections, and hence the axial extent of lamella breaks (**Figure 6**). For instance, an image sequence belonging to the aortic wall of the non-dilated zone of a 9 mo MFS ascending aorta showed several lamellae breaks (**Figure 6A**). Notice that throughout this sequence, distinct breaks appear, grow and end at diverse sites within the tunica media. Thus, topographically, a break starts as a small gap in one lamella (yellow box at slice 20 in **Figure 6A**), extends progressively in size to neighbouring lamellae, and finally affects most or all lamellae (see for example slice 340 in **Figure 6A**). This topographic break progression can also be easily visualised by 3D rendering (**Figure 6B-C**). In the supplementary movie, we display the crossing through the whole thickness of the tunica media by travelling through the lamellae breaks shown in **Figure 6**.

**Figure 7:**
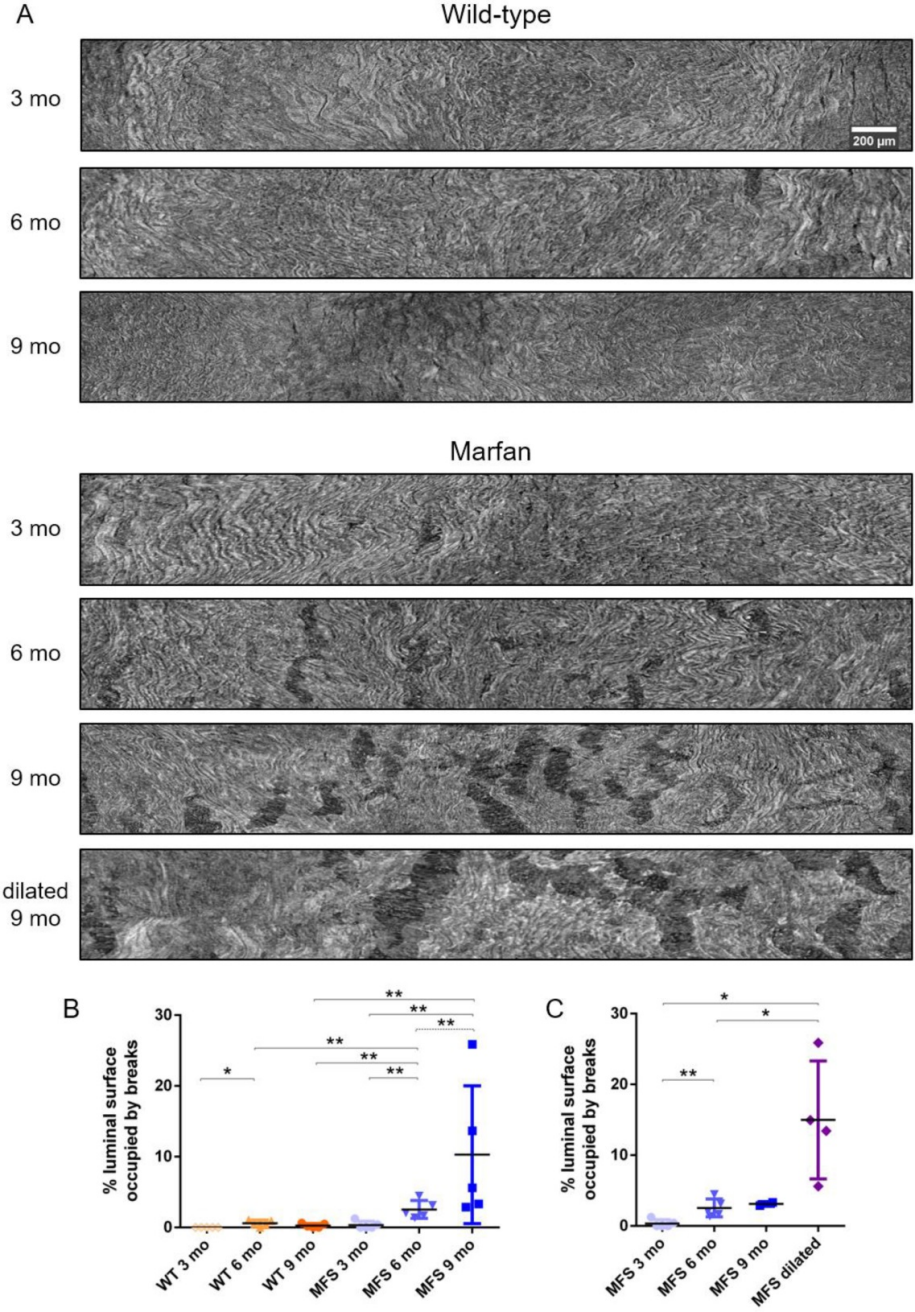
** Ascending aorta IEL luminal surface analysis of aged wild type and Marfan syndrome mice aortae. (A)** Representative images of the ascending aorta IEL surface. **(B)** Percentage of IEL surface occupied by breaks. **(C)** Percentage of the IEL surface occupied by breaks once Marfan 9 mo aortae were categorised into dilated (MFS dilated) or non-dilated (MFS 9 mo). WT: wild-type mice; MFS: Marfan syndrome mice. Results are mean ± SD. Continuous line corresponds to a mean statistical test, and dashed line to a dispersion statistical test. Scale bar, 200 µm.

To further analyse the damage progression in the MFS aortic wall, the IEL surface of ascending aortae was virtually generated in all samples to visualise the breaks occurring in the IEL in en-face view (**Figure 1D** and **Figure 7**). Example IEL surface images of WT and MFS aortae at differing ages are shown in **Figure 7A**. Note that IEL breaks are viewed as dark irregular discontinuities. Particularly, WT IEL surfaces were wavy and continuous regardless of age (**Figure 7A**). Occasionally, they also presented small breaks (**Figure 7B**). Conversely, MFS IEL surfaces usually showed several lamella breaks, whose number and size progressively increased with age (**Figure 7A**). Indeed, the total IEL surface space occupied by these breaks significantly increased with age (compare MFS 3, 6 and 9 mo in **Figure 7B**) and with the onset of a dilation (compare MFS 9 mo vs. MFS dilated 9 mo in **Figure 7A** and **Figure 7C**).

## Discussion

In this article, we demonstrate the ability of phase-contrast microCT to visualise and quantify large-scale vascular microremodelling because of age and inherited disease. We used a murine model of MFS (*Fbn1*^C1039G/+^) to visualise and analyse* ex vivo* the histopathological remodelling that takes place in the tunica media of the aortic wall. To achieve this aim, we utilized the high X-ray flux of synchrotron imaging, which facilitates rapid sample throughput. However, laboratory X-ray facilities can easily achieve similar resolution and contrast microCT scans, but at longer scan times 13. The obtained, aortic scans of middle-aged WT and MFS mice (3, 6 and 9 mo) were processed automatically by our in-house image processing GUI code to quantify a variety of structural features, and to provide highly informative 3D-based morphology images such as the ascending aorta IEL surfaces.

### Marfan-associated histopathological alterations in the ascending aorta tunica media as a potential model of an abnormally accelerated middle-ageing process

In contrast to other ageing studies that used older mice (24 months old) 37, our study allows some conclusions to be inferred about aortic wall remodelling in early and middle ages (3 to 9 mo, equivalent to 20 to 40 years old in humans 38). Both in WT and MFS aortae, we observed a gradual age-dependent increase in the mean values of luminal diameter, tunica media thickness, and media, lamellae and interlamellar space cross-sectional areas. Notably, remodelling of MFS aortae during age progression showed a similar tendency as in WT mice for most of the examined histomorphometric parameters, but by 9 mo (without categorising datasets), remodelling in MFS mice was significantly more pronouced than in WT 9 mo. In general terms, our results with the haploinsufficiency model of MFS (*Fbn1^C1039G/+^*) complement previous ones using a hypomorphic model (*Fbn1^+/mgΔ^*) 37, which together suggest that the genetic deficiency of fibrillin-1 seems to represent a pathological model of accelerated ageing in large arteries. In accordance with this idea, a recent report examining microRNA and mRNA expression profiles in blood samples of MFS patients 39 reported a reduced expression of protection of telomeres 1 (POT1) protein, favouring a reduced telomere length. This finding suggests an accelerated cardiovascular ageing. Moreover, POT1 was already established as a biomarker for thoracic aortic aneurysms and dissections 40,41. Our study provides new imaging insights in the analysis of structural remodelling in middle-aged MFS mice, which were not possible to assess by Faury *et al.* 37 using conventional histopathological techniques. In order to strengthen this tentative “accelerated ageing” concept, further studies would be needed to characterise biochemical and cellular markers of ageing in both middle-aged and truly aged mice.

Despite the apparent discrepancy in absolute values (compared with our study), it has been reported 42,43 a 1.5-fold increase in the tunica media thickness of MFS 8 mo compared to that of WT mice (~83 vs. ~55 µm, respectively; values deduced from their figures), which is close to our 1.2-fold increase between MFS and WT 9 mo (84 and 103 µm, respectively). Furthermore, Chung *et al.* 44 earlier published on the changes in elastic fibres area percentage in tunica media in mice and confirmed that lamellae area remained constant at ~50% in WT 3, 6 and 9 mo aortic arches, and that MFS non-aneurysmal thoracic aorta at 3 mo was similar to that of WT, but it decreased to ~44% in 6 and 9 mo mice. Notice that these values follow the same tendency and ratios as our results, but approximately 10 percentage points above, as our results showed constant WT lamellae area (42%) and MFS decreased from 40% at 3 mo to 31% at 9 mo. This difference between their absolute values and ours could be explained by the use of different imaging approaches (histological staining 42,44 vs. phase-contrast), which might differentially affect the dimensions of native lamellae. Moreover, regarding the previously mentioned changes in lamellae area, it should be stated that as yet, it is unclear which component(s) confer contrast to elastic lamellae in phase-contrast microCT imaging. Thus, taking into account that elastin synthesis is minimal in adulthood 2,3, we speculate that other lamellae components (not elastin), might be overexpressed in ageing and MFS, causing an increase in lamellae area. The future use of phase-contrast microCT in mice models genetically defective for fibrillins, fibulins, or other lamellae components will undoubtedly help to solve this question.

We highlight the heterogeneity in the MFS 9 mo datasets demonstrating that aortic damage did not occur at the same place for all individuals, even though they had the same *Fbn1* mutation, genetic background, and living conditions. This situation also happens in human beings, most likely because of the different genetic penetrance for the same *FBN1* mutation. Considering that this MFS 9 mo heterogeneity was mainly caused by aneurysm onset, we categorised the MFS datasets into non-dilated and dilated (MFS 3, 6, and 9 mo, and MFS dilated in **Figure 5**). This detailed analysis revealed that the media cross-sectional area increase in MFS middle ageing was a consequence of dilation development instead of age progression. Hence, cross-sectional area measurement stands as a more sensitive indicator of the remodelling of the aorta wall than the standard quantification of tunica media thickness.

Furthermore, although dilatation onset occurred mainly at 9 mo, significant aortic damage (IEL surface breaks) had already appeared at MFS 6 mo (**Figure 7**). These breaks resembled previously reported IEL cracks 2, which are a form of mechanically-induced damage. We suggest that these breaks/cracks start as a rupture between abnormally enlarged fenestrae 45, and progressively extend due to high tensile circumferential stresses 46. Therefore, our data of lamellae break/crack progression caused by tensile stress failure might be of interest to research groups working on computational modelling of aneurysms 47,48 and blood flow 49. In fact, the mechanical interpretation of this data might provide an explanation to previously published reports on the altered mechanical behaviour of MFS aortae 43,44,50. Moreover, these IEL breaks might be related to contrast agent infiltration within the aortic wall 51.

Although the differences in some histomorphometric parameters (WT ageing aortic diameter, media thickness and cross-sectional area; MFS progression lamella and interlamellar space areas, without dilated/non-dilated categorisation), did not reach the conventional minimal statistical significance (p≤0.05), they clearly demonstrated consistent trends, which does not necessarily mean that these structural remodelling events have no biological impact 52. In this sense, we are confident that our quantitative analyses are robust, as the measurements were obtained objectively using non-human-biased image analysis approaches applied to 200 images per sample (which is not the usual practise in conventional histology), and all datasets, except for MFS 9 mo, showed homogeneous average values with low statistical dispersion. However, it is also clear that sample size is a limitation and that future studies would benefit from a larger sample size to provide greater statistical power.

Another limitation of the study is the use of classical histological tissue processing methods (paraformaldehyde fixation, dehydration and paraffin embedding). Although their use allows for comparative analysis with previous works 53,54, it is possible that dehydration may differentially affect WT and MFS mice vessels since the MFS aortic wall is enriched in hydrophilic proteoglycans (which are commonly localised at elastic lamellae breaks 55), and therefore it might be differently affected than the WT aortic wall.

### Potential of vascular tissue imaging by phase-contrast microCT

X-ray phase-contrast microCT imaging has great potential to visualise soft tissues, providing new structural information and increasing understanding of healthy vessel function and disease progression 18. For example, unlike conventional transverse histological sections, phase-contrast microCT can provide an image of the aorta IEL surface 15 at high resolution and with a large field of view 14,17,51. It also avoids artefacts induced by sample staining or sectioning. Although here we used *ex vivo* synchrotron-based microCT imaging, laboratory microCT devices are already capable of high-resolution acquisition (although with longer scanning times) 15. Moreover, microCT devices are currently capable of *in vivo* vascular imaging 56-58, and further instrumental improvement may facilitate higher resolution scans that permit visualisation of vascular microarchitecture *in vivo*. Therefore, going forward, examination of IEL surface damage by microCT might be a novel and feasible approach to early diagnosis of ascending aorta aneurysm in preclinical research and the measurement of aortic wall structural features might serve to assess ageing progression. It may be that in the near future these characteristics of the aorta could be monitored *in vivo* by microCT, providing new structural information at high-resolution and in real time about ageing and/or aneurysm progression, complementing the current echocardiographic-based imaging techniques used in clinical diagnosis and investigation 59. In this direction, we have already shown that microCT is capable of visualising native ECM-rich tissues 23. Hence, it will be possible shortly to characterise 3D microscale remodelling in dynamically changing vessels due to *in vivo* variations in intra-luminal pressure.

## Conclusion

We have demonstrated that high-resolution phase-contrast microCT is a valid and potent technique for the study of vascular tissues, providing both transverse and 3D perspectives. This imaging approach overcomes the limits imposed by conventional histological methods such as small size, staining and destructive sectioning of samples. Furthermore, automatic computational measurement of structural features on multiple images stands is a non-biased robust method for studying in detail histological remodelling processes. We have applied the implemented methodology to the study of middle-aged WT and MFS mice (3, 6 and 9 months old) ascending aortae. Measurement of key histological parameters in the aortic wall suggests that tissue remodelling in the MFS vessel resembles an accelerated normal ageing process. These age-associated changes are exacerbated in abnormally dilated zones of the ascending aorta, but media thickness is not altered. In addition, notable IEL breaks occurred months before the onset of the dilation. In summary, high-resolution microCT is an excellent option for vascular imaging, and allows the acquisition of highly detailed entire vessel three-dimensional scans, providing volumetric data to assess submicron arterial wall remodelling. Application of the technique to non-chemically fixed tissues 23 would avoid inducing potential artefacts during specimen preparation and pave the way for future clinical diagnosis.

## Supplementary Material

Supplementary methods and figures.

Supplementary movie 1.

## Abbreviations

GUI: graphical user interface
IEL: internal elastic lamina
MFS: Marfan syndrome
microCT: X-ray computed micro-computed tomography
mo: months old
WT: wild-type.
