# Supplementary material for: MicroCT imaging reveals differential 3D micro-scale remodelling of the murine aorta in ageing and Marfan syndrome
Source: Theranostics. 2018 Nov 15;8(21):6038–52. doi: 10.7150/thno.26598 (PMC6299435; doi:10.7150/thno.26598)
Supplement: Supplementary file 1 — Supplementary methods and figures. [file thnov08p6038s1.pdf]

SUPPLEMENTAL MATERIAL

MicroCT Imaging Reveals Differential 3D Micro-Scale Remodelling Of The Murine Aorta In Ageing And Marfan Syndrome

Júlia López-Guimet, Lucía Peña Pérez, Robert S. Bradley, Patricia García-Canadilla, Catherine Disney, Hua Geng, Andrew Bodey, Philip J. Withers, Bart Bijnens, Michael J. Sherratt, Gustavo Egea

SUPPLEMENTARY METHODS

Segmentation of aortic wall and lumen

Before thresholding, the contrast of the image was automatically adjusted using the Matlab function “imadjust”. Then, the gradient magnitude of the image was calculated using Sobel filter and subsequently smoothed with a Gaussian filter (sigma=3 pixels). The filtered gradient image was finally binarised using a threshold automatically determined by the Matlab function “graythresh” which uses Otsu's method to minimise pixel's intraclass variance. Finally, some morphological operations were applied to clean up the segmentation. An illustrative example of the different segmentation steps is shown in **supplementary Figure 2**.

Segmentation of lamellae

After adjustment of the image contrast by imadjust, a median filter was applied to remove some of the noise present in the images. Then, an adaptive thresholding method implemented in Matlab (“adaptthresh”) was used to automatically segment the lamellae. This method uses a large-neighbourhood mean filter and, in our case, we used a Gaussian filter with a sensitivity factor of 0.45 to locally compute the threshold. The sensitivity factor was determined empirically by testing several values. The same parameters were used for both WT and MFS groups. Finally, in order to clean up the segmentation, small elements (with a size less than 30 pixels) were removed using the Matlab function “bwareaopen”. **Supplementary Figure 3** shows an illustrative example of the lamellae segmentation procedure.

SUPPLEMENTARY FIGURES

Supplementary Figure 1

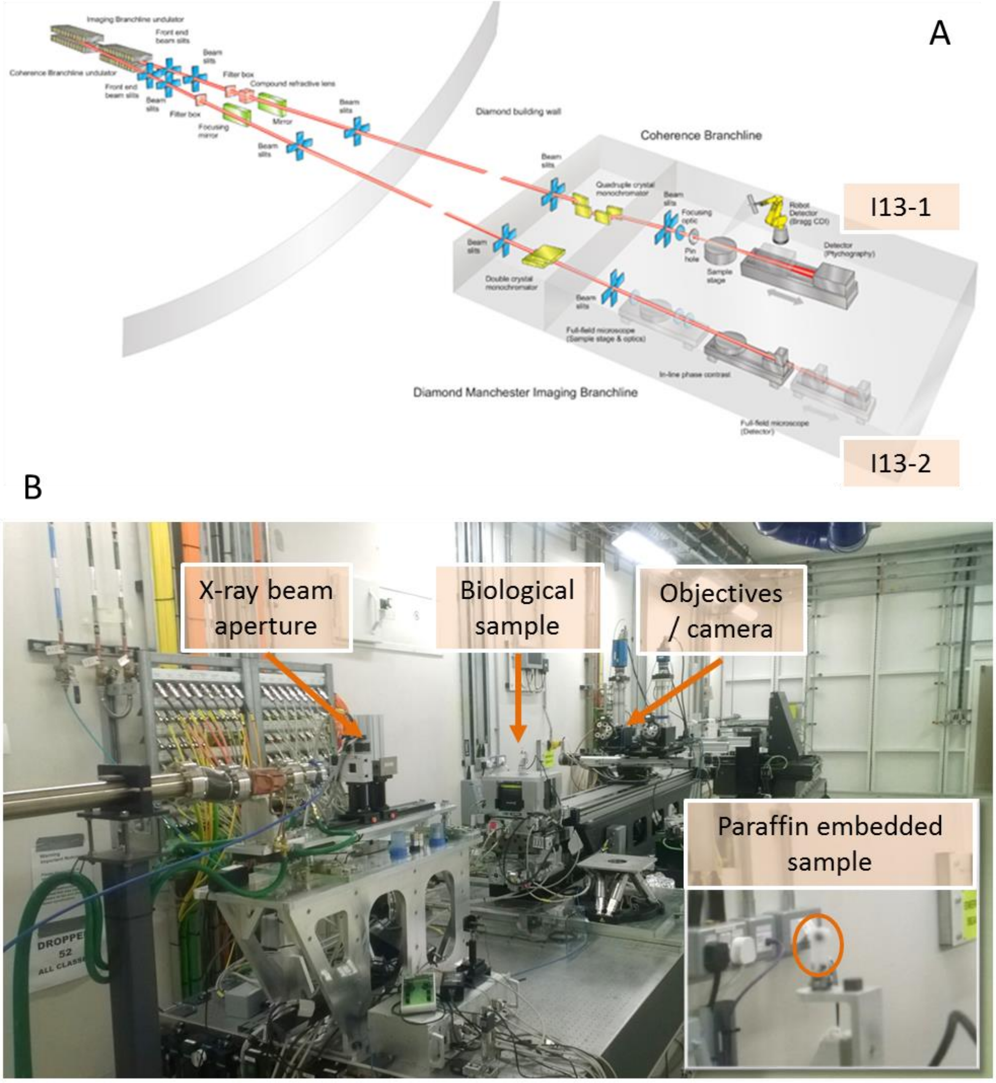

Supplementary figure 1. Phase contrast imaging at the I13 Diamond synchrotron beamline. (A) The 250 m long I13 beamline is split into two independent branchlines supporting coherent X-ray diffraction (I13-1) and 8-30 keV in-line phase contrast tomography over a large field of view (I13-2). Imaging in this study was performed on the I13-2 branchline. **(B)** In the experimental "hutch", the sample is mounted on a rotating table between the X-ray source and the scintillation screen/optical objective and CCD camera. The paraffin-embedded sample can be more clearly seen in the magnified inset.

Supplementary figure 2

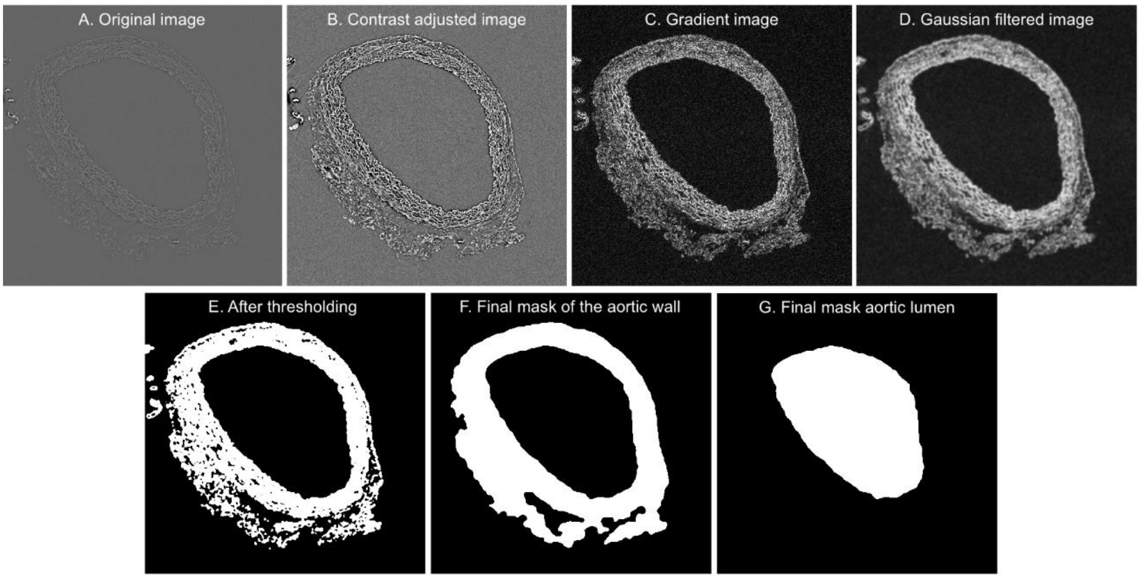

Supplementary figure 2. Segmentation protocol of the aortic wall from a microCT scan. **(A)** Original image of the aortic wall surrounded by paraffin. **(B)** Adjusted contrast image. The paraffin texture is more homogeneous than the aortic tissue texture. **(C)** Gradient image obtained after applying a Sobel filter. **(D)** Smoothed image by Gaussian filter. **(E)** Binary image obtained by thresholding. **(F)** Mask of the aortic wall generated by closing small gaps between white isles. **(G)** Lumen mask of the aorta.

Supplementary figure 3

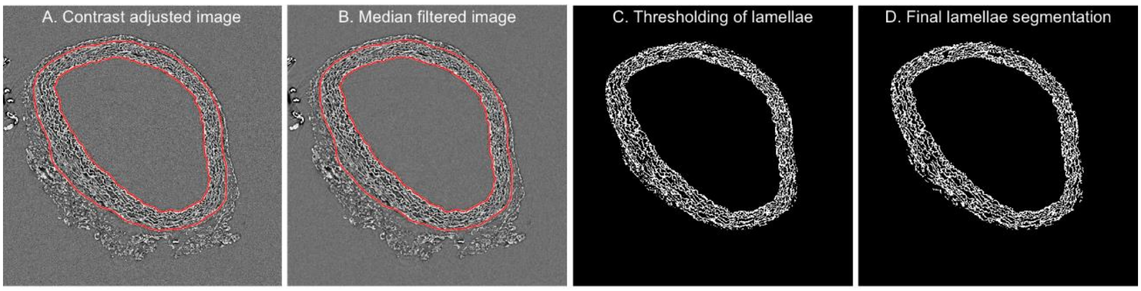

Supplementary figure 3. Illustrative example of the lamellae segmentation process. (A) Image after contrast adjustment. **(B)** Denoised image after filtering with a median filter. **(C)** Binary mask after performing the automatic thresholding using a local adaptive threshold with a sensitivity factor of 0.45 and a Gaussian weighted mean to calculate the local threshold. **(D)** Final lamellae segmentation after removing small objects.

Supplementary movie

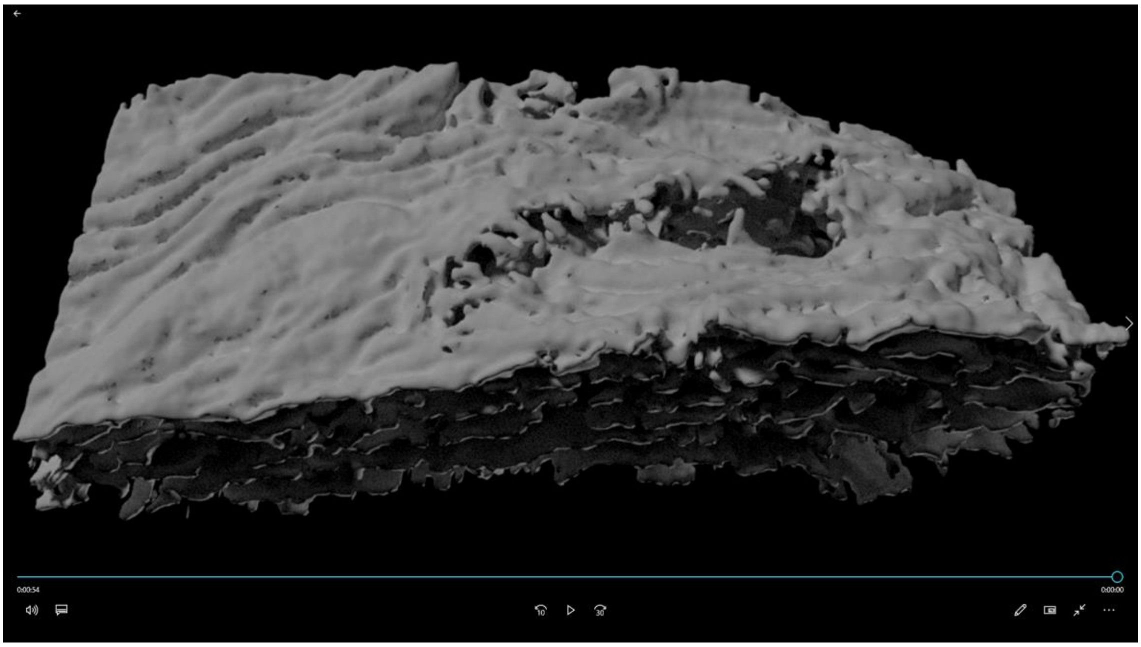

Supplementary movie. 3D volume render of a portion of aortic tunica media; only lamellae are rendered (white objects). A break affecting all lamellae is observed, and the camera can travel through it from the luminal side to the adventitial side.
